# Anodal tDCS alters perceived visual contrast in humans

**DOI:** 10.1016/j.isci.2026.118005

**Published:** 2026-10-27

**Authors:** Yosun Yoon, Sang Wook Hong

**Affiliations:** 1 Department of Psychology, Florida Atlantic University, Boca Raton, FL 33431, USA; 2 Stiles-Nicolson Brain Institute, Florida Atlantic University, Boca Raton, FL 33431, USA

**Keywords:** contrast, appearance, transcranial direct current stimulation, tDCS

## Abstract

Cortical gain modulation is thought to alter perceptual experience, most prominently demonstrated by the effects of spatial attention on contrast appearance. Whether externally induced changes in cortical excitability produce comparable perceptual consequences remains unclear. Here, we tested whether anodal transcranial direct current stimulation (tDCS) over occipital cortex alters perceived contrast. Using a contrast-appearance paradigm, participants judged which of two gratings appeared higher in contrast. Anodal stimulation produced systematic shifts in the point of subjective equality (PSE), such that stimuli presented in the contralateral visual field were perceived as higher in contrast. This effect reversed across stimulus configurations and was absent under sham stimulation, ruling out response bias. Psychometric slopes did not change significantly, consistent with stable discrimination precision around the PSE. These findings demonstrate that tDCS alters contrast appearance, suggesting that changes in cortical excitability can directly influence perceptual experience through gain-like modulation of sensory responses.

## Highlights

• Anodal tDCS targeting right occipital cortex altered perceived contrast
• The effect reversed with stimulus configuration, indicating spatial specificity
• Sham stimulation produced no reliable change in contrast appearance

## Graphical abstract

**Figure:**
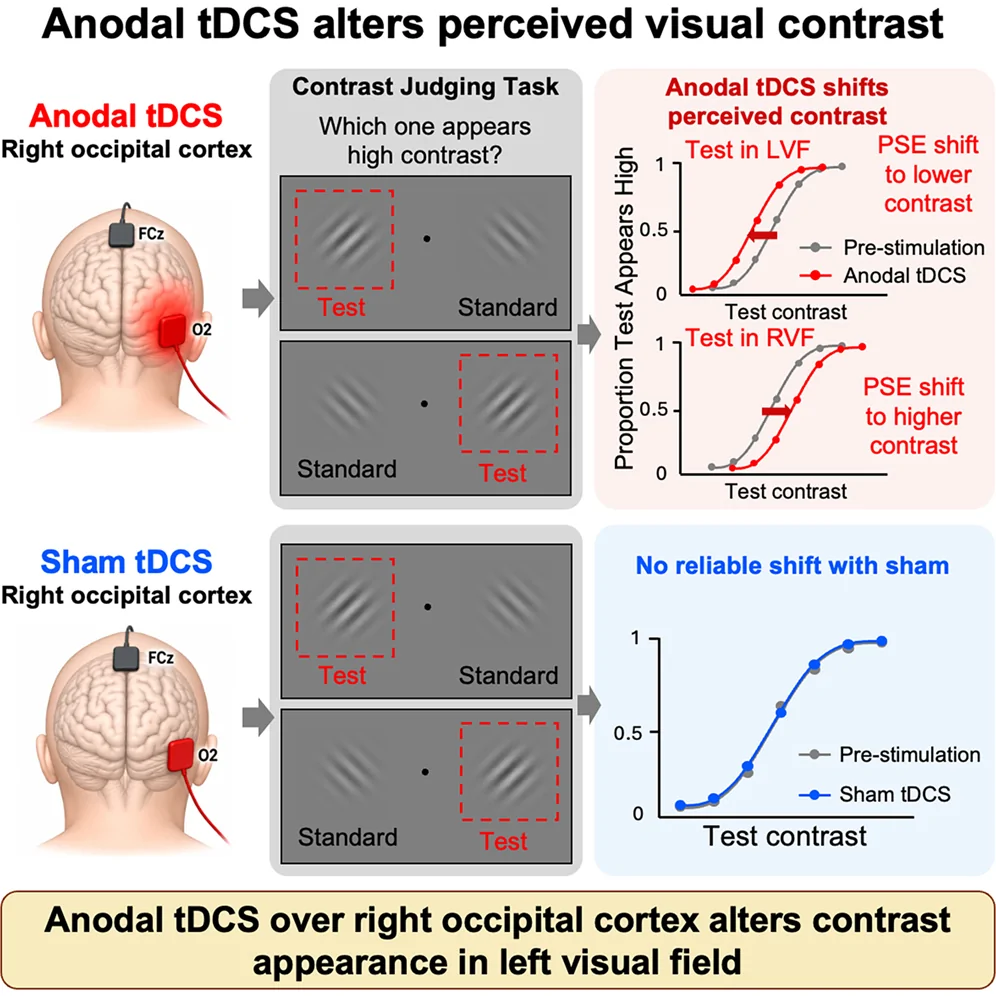

## Teaser

Natural sciences; Biological sciences; Neuroscience; Cognitive neuroscience

## Introduction

Contrast is a fundamental aspect of visual information that determines object visibility, shapes feature salience, and plays a critical role in visual processes such as object recognition and motion perception. For example, regions around eyes generally have the highest contrast within a face, and therefore the eyes become the most salient and informative facial feature.^1^ Neural responses to visual stimuli are monotonically related to the contrast of the stimuli, and in turn, determine the strength of perceptual experiences. A central question in vision science is how such contrast-dependent changes in neural activity shape perceptual experience beyond mere changes in detection sensitivity.

Visual attention has been extensively studied as a major modulator of neural responses to visual stimuli. A large body of research suggests that covert spatial attention enhances visual processing by demonstrating that attention improves contrast sensitivity across psychophysical^2^^,^^3^^,^^4^^,^^5^ and neurophysiological (for review, see Reynolds and Chelazzi^6^) measures and is associated with enhanced neural responses in multiple levels of visual information processing. Importantly, attention does not merely improve detectability. Using a contrast-appearance paradigm, Carrasco, Ling, and Read^7^ demonstrated that directing covert attention to a stimulus increases its perceived contrast. Observers judged attended stimuli as higher in contrast than physically identical unattended stimuli. Neurophysiological evidence suggests that these appearance shifts arise from gain modulation in the early visual cortex, typically characterized as a contrast or response gain enhancement.^8^^,^^9^ Psychophysical modeling further supports this, demonstrating that such gain mechanisms effectively increase the perceived strength of attended stimuli.^10^ Together, these findings highlight that modulating neural responses in early visual cortex can alter both contrast sensitivity (detection thresholds) and contrast appearance (suprathreshold experience).

Given that endogenous factors like attention can modulate neural responses and perceptual experiences, there has been growing interest in whether exogenous tools, such as transcranial direct current stimulation (tDCS), can similarly alter cortical excitability and visual processing. TDCS has been proposed to modulate cortical excitability by altering neuronal membrane potentials.^11^ Anodal stimulation over motor cortex is typically associated with acutely increased excitability^12^^,^^13^ and reduced GABAergic inhibition.^11^^,^^14^ In the domain of visual perception, anodal tDCS over the occipital cortex has been associated with reduced phosphene thresholds,^15^ and greater amplitudes in visual-evoked potentials.^16^ Although anodal tDCS is generally associated with increased cortical excitability, accumulating evidence indicates that its behavioral effects are often heterogeneous across studies. Recent reviews and meta-analyses have highlighted substantial inter-individual variability and task-dependent outcomes, with stimulation effects influenced by factors such as baseline cortical state, stimulation parameters, and the behavioral measures employed.^17^^,^^18^^,^^19^ Consequently, the behavioral consequences of occipital tDCS remain incompletely understood, raising the possibility that differences in task sensitivity may contribute to the heterogeneous findings reported across studies.

This possibility is particularly relevant for studies of visual contrast processing. Most previous work has assessed whether anodal tDCS improves visual performance by measuring contrast sensitivity. Although some studies reported facilitation,^20^^,^^21^^,^^22^ others found non-significant^23^^,^^24^ or polarity-reversed effect.^25^ Furthermore, individual baseline performance modulates stimulation efficacy,^26^ suggesting that cortical state critically shapes outcomes. One potential source of this inconsistency is that nearly all previous studies have relied on detection thresholds as the primary behavioral outcome. Yet, if anodal tDCS enhances cortical gain in a manner analogous to spatial attention, its most direct perceptual consequence may be altered contrast appearance rather than altered sensitivity. Despite this theoretical implication, it remains unknown whether anodal tDCS changes the perceived contrast of suprathreshold stimuli. The present study addresses this gap by adapting the contrast-appearance paradigm developed by Carrasco et al.^7^ Rather than measuring detection thresholds, we directly assessed whether anodal stimulation over visual cortex alters the perceived contrast of a grating stimulus. If anodal tDCS increases the effective strength of visual responses, stimuli represented in the stimulated hemisphere should appear higher in contrast, producing a systematic shift in PSE. Such a finding would suggest that externally induced modulation of cortical excitability can influence subjective visual appearance in a manner functionally analogous to attentional modulation of visual processing.

By dissociating appearance from sensitivity, this study provides a new test of how neuromodulation shapes visual perception. Demonstrating that anodal tDCS alters contrast appearance would not only clarify the mixed literature on contrast sensitivity but also extend models of perceptual gain control beyond visual attention to include externally induced cortical modulation.

## Results

### Experimental design and predicted effects on contrast appearance

Participants judged which of two simultaneously presented Gabor patches appeared higher in contrast while the contrast of a test stimulus was varied across trials (Figures 1A and 1B). The standard stimulus (15.49% contrast) was presented either left or right visual field randomly, and the test stimulus with various contrast was presented the other side of the visual field (see STAR Methods for details). tDCS was applied to only right occipital cortex (Figure 1C), and we expected that the stimulus presented in the left visual field (LVF) would be affected by tDCS. Before presenting the data, it would be useful to consider how different perceptual effects would be expressed in the psychometric function (Figure 2). A change in contrast appearance would produce a horizontal shift in the point of subjective equality (PSE) as demonstrated in Carrasco et al.,^7^ whereas a change in discrimination precision would alter the steepness of the psychometric function around the PSE. In the context of the present suprathreshold appearance task, the psychometric slope reflects the precision with which observers discriminate small differences in perceived contrast around the PSE, rather than contrast sensitivity in the conventional vision science sense, which typically refers to detection-threshold performance. Thus, a PSE shift with little change in slope would indicate altered appearance while discrimination precision remains relatively stable, whereas a change in slope with little or no PSE shift would indicate altered discrimination precision.

**Figure 1:**
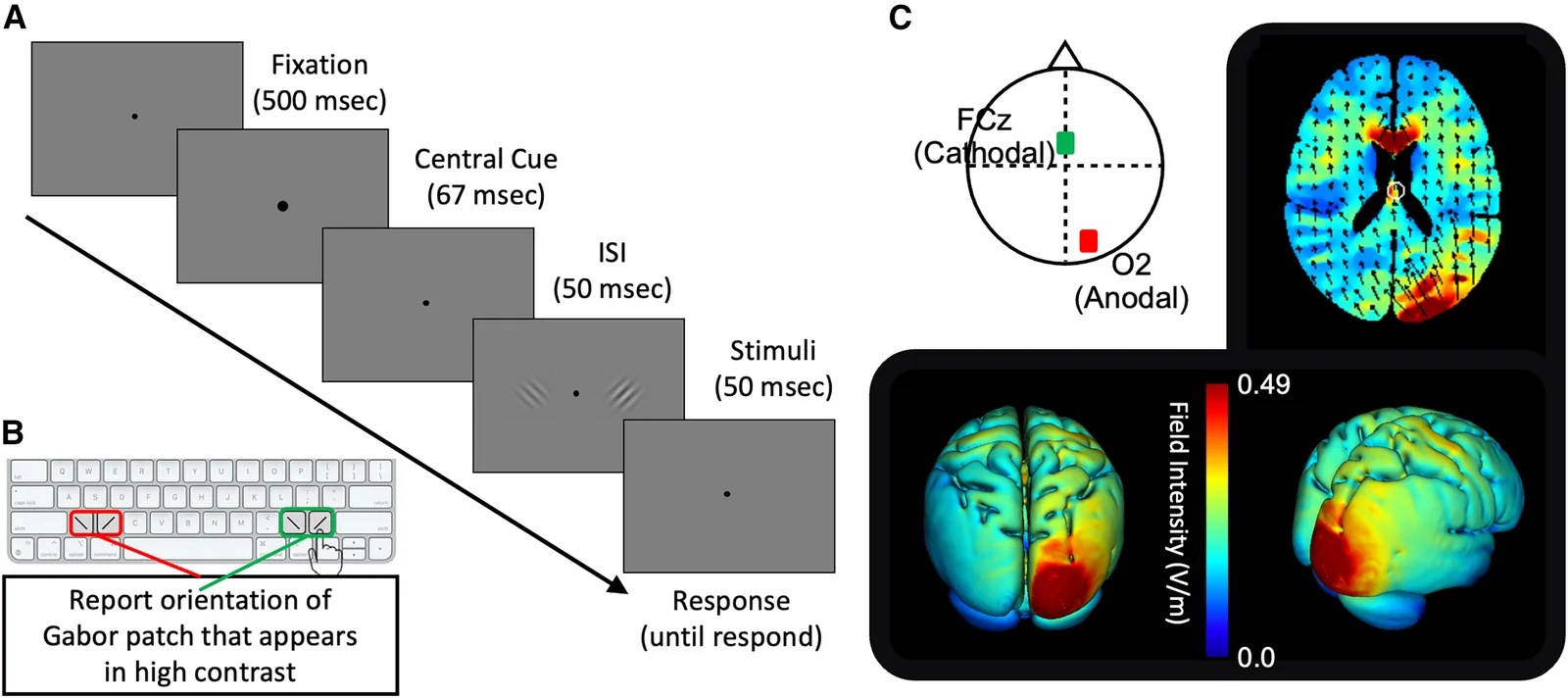
Experimental design and stimulation protocol (A) Sequence of events within a trial. Each trial began with fixation (500 ms), followed by a centrally presented cue (67 ms) and a brief interstimulus interval (50 ms). Two Gabor patches were then presented simultaneously (50 ms). Participants subsequently reported the orientation (left- or right-tilted) of the Gabor patch that appeared higher in contrast, rather than indicating its location. (B) Response mapping. Participants indicated the orientation (left- or right-tilted) of the Gabor patch that appeared higher in contrast using designated keyboard responses, following the procedure of Carrasco et al.^7^ (C) tDCS montage and modeled electric field distribution. Anodal stimulation was applied over the right occipital cortex (O2), with the cathodal electrode placed over the fronto-central site (FCz). The accompanying maps illustrate the simulated electric field intensity (V/m), showing peak modulation over occipital regions.

**Figure 2:**
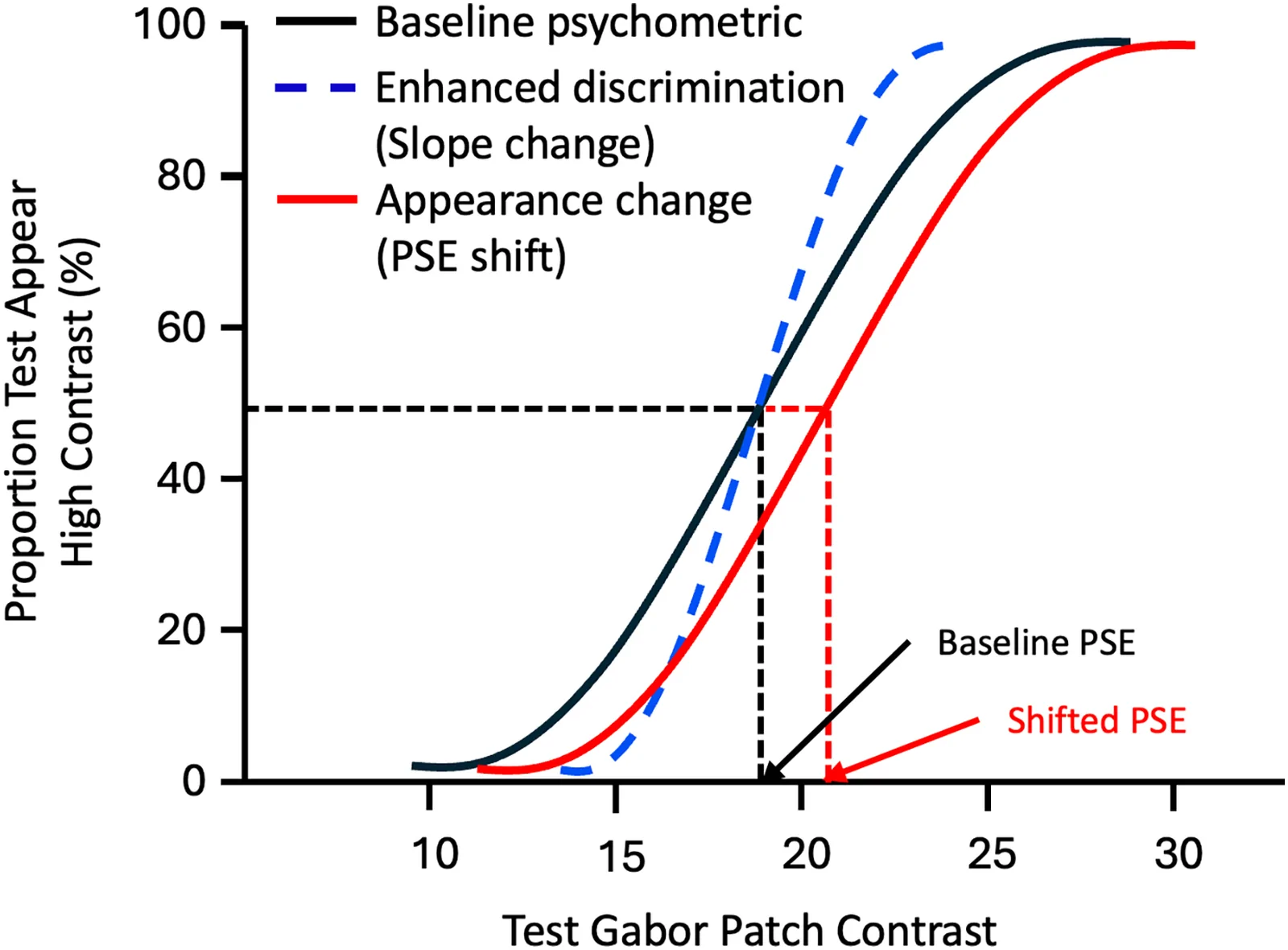
Predicted effects of tDCS on psychometric functions A horizontal shift of the psychometric function reflects a change in the PSE, indicating altered contrast appearance without necessarily changing discrimination precision (red solid line). In contrast, a change in psychometric slope with little or no change in PSE indicates altered discrimination precision around the PSE (blue dashed line). The present study predicted and observed the pattern illustrated by the appearance-shift model

For each participant and condition (anodal tDCS and sham stimulation conditions), we computed the proportion of trials in which the test stimulus was judged to appear higher in contrast as a function of test contrast level (9 target contrast levels; 20 repetitions per level). We fit psychometric Weibull sigmoid functions using the Bayesian inference framework implemented in the MATLAB toolbox psignifit^27^ (version 3). The PSE was estimated from the fitted psychometric function as the test contrast at which participants reported the test stimulus as higher in contrast than the standard on 50% of trials. PSE shifts were quantified as the within-participant difference between stimulation and pre-stimulation PSEs (ΔPSE = PSE_stim_ – PSE_pre_). When the standard stimulus was presented in the LVF (left standard right test, LSRT), it was expected that PSE_stim_ would shift rightward from PSE_pre_ (positive ΔPSE) indicating that a higher physical test contrast was required to match the standard during stimulation (consistent with increased perceived contrast of the standard stimulus). Conversely, when the test stimuli were presented in the LVF (left test right standard, LTRS), it was expected that PSE_stim_ would shift leftward from PSE_pre_ (negative ΔPSE) indicating that a lower physical test contrast was required to match the standard during stimulation (consistent with increased perceived contrast of the test stimuli).

### Anodal tDCS shifts perceived contrast in the predicted direction

Figure 3 shows the averaged contrast-appearance judgment and fitted psychometric functions to the averaged data. As predicted, we found that PSE measured during anodal tDCS stimulation shifted rightward from the pre-stimulation baseline when the standard stimulus was presented in the LVF (Figure 3A) and shifted leftward when the standard stimulus was presented in the right visual field (Figure 3C). This pattern of shift in PSE was not observed in sham control conditions (Figures 3B and 3D). Examination of individual-level fits showed that the psychometric functions fit the data well for all participants. This finding indicates that the perceived contrast of the stimuli was heightened reliably among participants due to anodal tDCS applied in the contralateral hemisphere.

**Figure 3:**
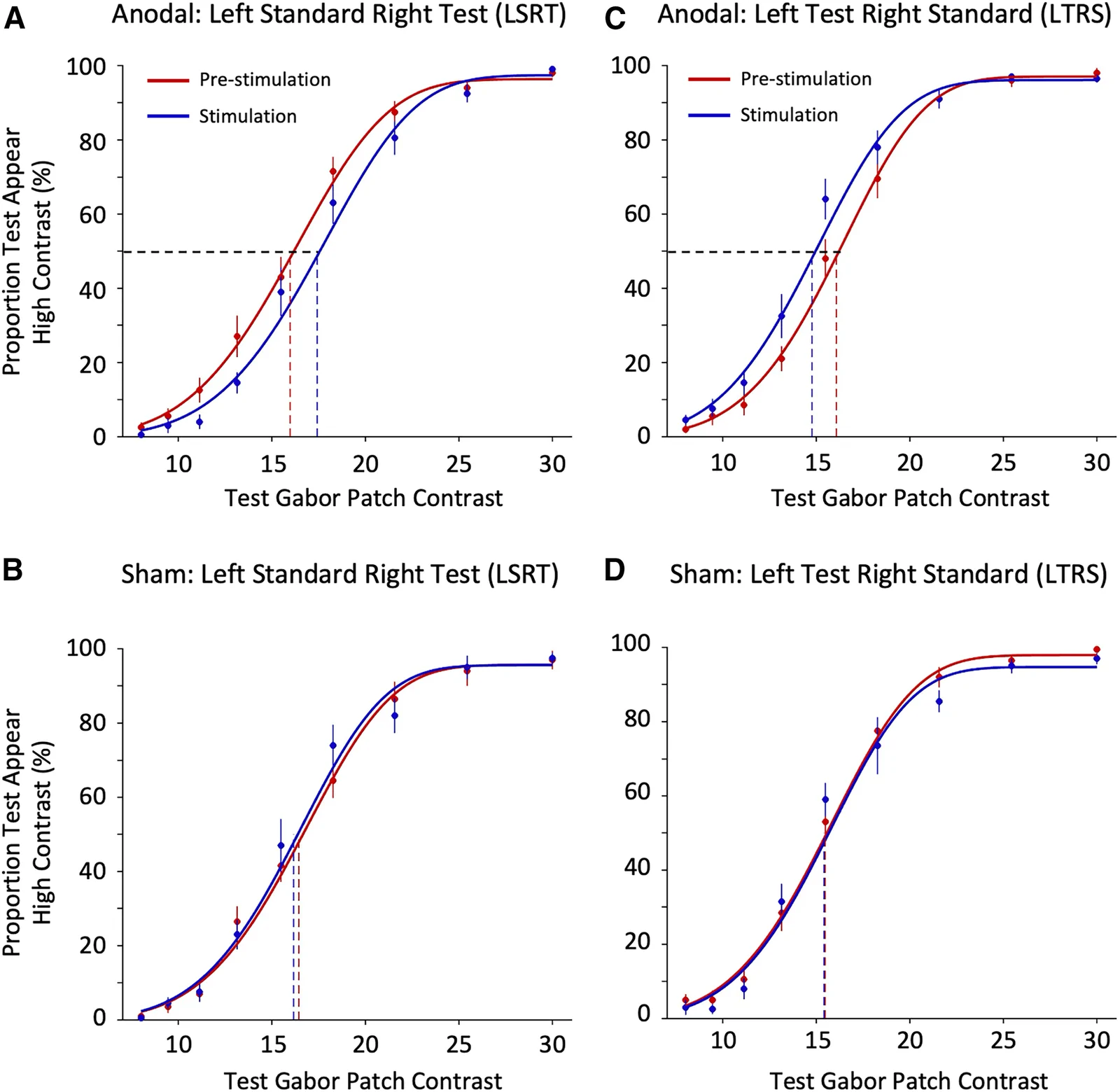
Averaged contrast-appearance judgments and fitted psychometric functions (A) Anodal tDCS with LSRT condition, in which the standard stimulus was presented in the LVF and the test stimulus in the right visual field (RVF). (B) Sham control with LSRT condition. (C) Anodal tDCS with LTRS condition, in which the standard stimulus was presented in the RVF and the test stimulus in the LVF. (D) Sham control with LTRS condition. Points represent the mean proportion of trials in which the test stimulus was judged to appear higher in contrast, plotted as a function of test contrast. Symbols represent mean responses across participants (*N* = 10) and error bars indicate ±1 SEM. Solid lines show fitted psychometric functions (red = pre-stimulation; blue = stimulation). Vertical dashed lines indicate the estimated PSE (50% point), and the horizontal dashed line marks chance level (50%).

### Anodal tDCS produces configuration-dependent shifts in perceived contrast

To confirm the shift in perceived contrast is robust and statistically reliable, we first examined the signed change in PSE (ΔPSE), in which shifts in the predicted direction for each configuration (LTRS and LSRT) were aligned. A paired-samples *t* test revealed that the tDCS-induced shift in perceived contrast was significantly greater during anodal stimulation than during sham (t[9] = 4.44, *p* = 0.0016, Cohen’s d_z = 1.403). Note that the result of statistical analysis was comparable when the author participant was excluded from the analysis (t[8] = 6.47, *p* = 0.0002, Cohen’s d_z = 2.156) and when one outlier was excluded from the analysis (t[8] = 4.442, *p* = 0.0022, Cohen’s d_z = 1.474). As shown in Figure 4A, anodal stimulation produced a robust positive shift in perceived contrast (M = 1.64%, SE = 0.33), whereas the sham condition did not (M = −0.33%, SE = 0.27). The 95% confidence interval for the difference between conditions was [0.97%, 2.98%]. A one-sample *t* test confirmed that the signed ΔPSE under anodal stimulation was significantly greater than zero, t(9) = 4.96, *p* = 0.0008, 95% CI [0.89%, 2.39%], indicating a reliable shift in perceived contrast in the predicted direction. In contrast, the sham condition did not differ from zero, t(9) = −1.23, *p* = 0.25, 95% CI [−0.94%, 0.28%].

**Figure 4:**
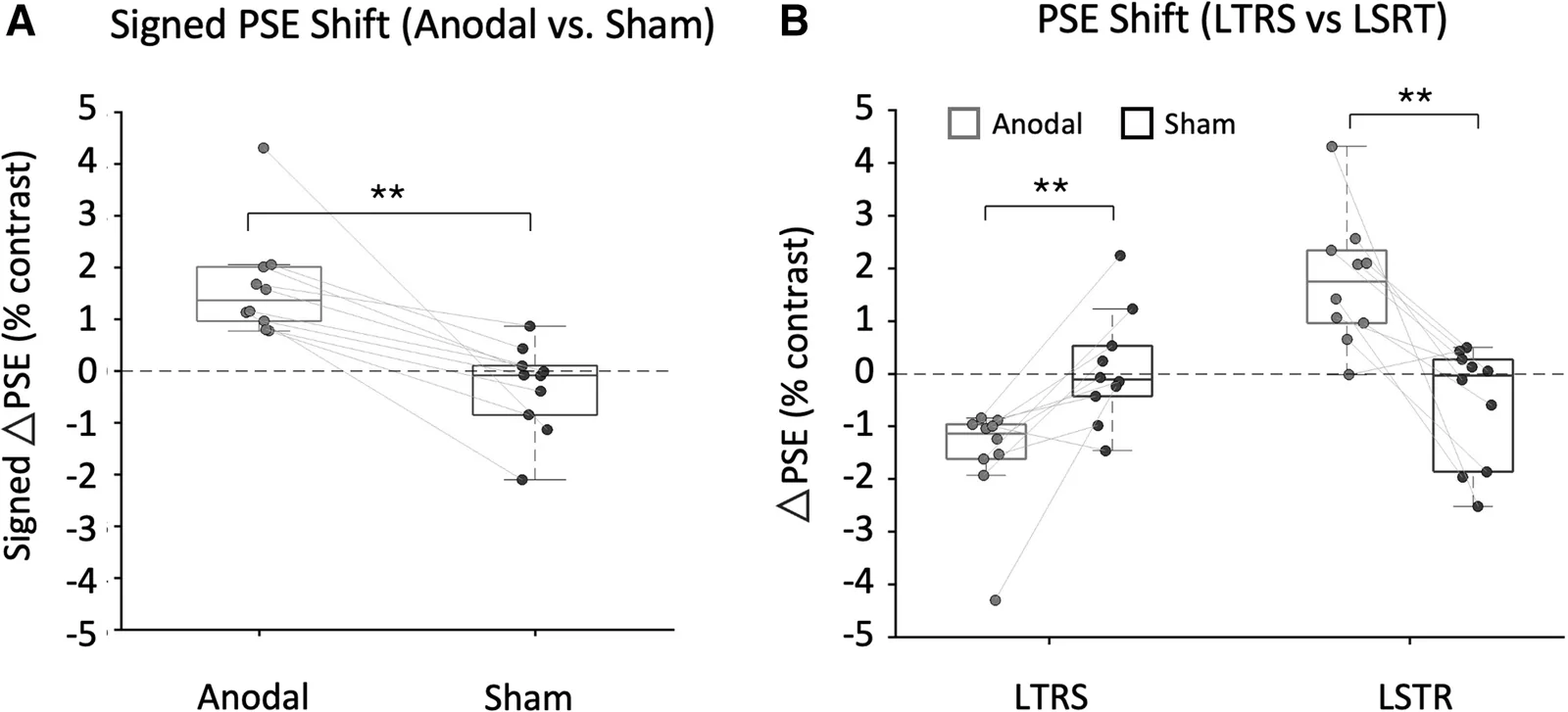
Effects of anodal tDCS on perceived contrast (A) Signed change in the PSE comparing anodal and sham stimulation. Positive values indicate a shift in perceived contrast in the predicted direction (i.e., stimuli presented in the stimulated hemifield appearing higher in contrast). (B) Raw ΔPSE values plotted separately for LTRS and LSRT configurations. Anodal stimulation induced opposite shifts in the two configurations (negative for LTRS, positive for LSRT), consistent with a configuration-dependent modulation of contrast appearance, whereas no reliable difference was observed under sham condition. Boxes indicate the interquartile range, with the horizontal line representing the median; whiskers denote the range excluding outliers. Individual data points represent participants (*N* = 10), and connecting lines indicate paired measurements from the same participant. The dashed horizontal line indicates no change (ΔPSE = 0). Statistical comparisons indicated by brackets were performed using two-tailed paired-samples *t* tests. In (A), signed ΔPSE was compared between anodal and sham stimulation. In (B), anodal and sham stimulation were compared separately within the LTRS and LSRT configurations. Asterisks indicate *p* < 0.01.

To examine the full pattern of effects, we conducted a 2 (stimulation: Anodal, Sham) × 2 (configuration: left-standard-right-test [LSRT], left-test-right-standard [LTRS]) repeated-measures ANOVA on raw ΔPSE values (Figure 4B). There was a significant main effect of configuration, F(1, 9) = 10.36, *p* = 0.0105, ηp^2^ = 0.535, indicating overall differences between LTRS and LSRT conditions. Critically, this main effect was qualified by a significant stimulation × configuration interaction, F(1, 9) = 19.69, *p* = 0.0016, ηp^2^ = 0.686. This interaction reflects the predicted sign reversal in ΔPSE across configurations under anodal stimulation. The main effect of stimulation conditions was not significant, F(1, 9) = 1.47, *p* = 0.256, consistent with the expectation that stimulation effects depend on visual field configuration rather than producing a uniform shift. Planned comparisons confirmed that under anodal stimulation, ΔPSE differed significantly between configurations, t(9) = −4.96, *p* = 0.0008, 95% CI [−4.78%, −1.79%], indicating opposite shifts in LTRS (M = −1.54%) and LSRT (M = 1.75%) conditions. No such difference was observed in the sham condition, t(9) = 1.23, *p* = 0.25. Direct comparisons between stimulation conditions further showed that anodal tDCS significantly modulated perceived contrast relative to sham in both configurations: for LTRS, t(9) = −3.68, *p* = 0.0050, 95% CI [−2.63%, −0.63%], and for LSRT, t(9) = 3.85, *p* = 0.0039, 95% CI [0.96%, 3.68%].

### Anodal tDCS does not significantly alter psychometric slope

Analyses of the psychometric slope revealed no significant effects of stimulation. Neither the primary comparison between anodal and sham stimulation, t(9) = −1.05, *p* = 0.32, nor the repeated-measures ANOVA showed any main effects or interactions (all *p*s > 0.32). This indicates that tDCS did not reliably alter the steepness of the psychometric function, suggesting that the observed effects on PSE reflect changes in perceived contrast rather than changes in contrast sensitivity.

## Discussion

In the current study, we demonstrated that externally enhanced neural excitability can result in altering perceptual experience. The grating presented in the visual field corresponding to the early visual cortex where anodal tDCS was applied appeared higher contrast than the one presented in the opposite visual field. This modulation of contrast appearance due to anodal tDCS was not observed in sham control condition, indicating that the appearance shift is likely attributable to increased cortical excitability induced by anodal tDCS. These results demonstrate that anodal tDCS over early visual cortex produces a reliable and direction-specific modulation of perceived contrast, whereas comparable effects were not consistently observed in measures in sensitivity.

The critical stimulation × configuration interaction provides strong evidence that the observed effects are not simply due to nonspecific changes in arousal or decision bias. Instead, the direction of the PSE shift depended on which stimulus (target vs. standard) was presented in the visual field corresponding to the stimulated hemisphere. This sign reversal is a hallmark of a change in contrast appearance and mirrors the logic used in attention studies to dissociate perceptual changes from response bias. Such spatial specificity suggests that anodal tDCS selectively enhances processing in the targeted cortical region, consistent with models in which stimulation increases the responsiveness of neuronal populations representing the contralateral visual field. This interpretation aligns with previous reports that tDCS effects depend on baseline neural state and task demands.^23^

One potential concern is whether intrinsic differences between the left and right visual fields could account for the current findings. Previous psychophysical studies have generally found comparable contrast sensitivity across visual fields with no consistent evidence for systematic hemifield differences.^28^^,^^29^ Likewise, Carrasco et al.^7^ randomized presentation of the standard and test stimuli across the two visual fields in their contrast-appearance paradigm and did not report systematic visual-field asymmetries in perceived contrast. More importantly, a fixed visual-field bias cannot readily explain the present results because the direction of the PSE shift reversed depending on whether the standard or test stimulus occupied the stimulated visual field, whereas no corresponding pattern was observed during sham stimulation. This configuration-dependent sign reversal strongly supports the interpretation that the observed changes arose from stimulation-induced modulation of cortical processing rather than from an inherent visual-field asymmetry.

Although the behavioral results demonstrated clear spatial specificity, this should not be interpreted as evidence that the neural effects of stimulation were confined exclusively to the right occipital cortex. The sign reversal observed across the two stimulus configurations indicates that tDCS-induced effect preferentially influenced visual processing in the contralateral visual field. However, conventional sponge-electrode tDCS produces relatively diffuse current distributions, and the montage used here was designed to target the right occipital cortex rather than exclusively stimulate that region alone. Consequently, the present findings should be interpreted as demonstrating that a right occipital-central frontal stimulation montage systematically altered perceived contrast, rather than establishing the precise cortical locus responsible for the behavioral effect. Future studies combining individualized electric-field modeling with higher focality stimulation approaches, such as HD-tDCS, may further clarify the neural substrates underlying these perceptual changes. It should also be noted that the electric-field model shown in Figure 1C was generated using a standard template head rather than individualized anatomical MRI data. Because skull thickness, cerebrospinal fluid volume, and cortical anatomy vary substantially across individuals, the actual current distribution likely differed between participants. Consequently, the modeled electric field should be regarded as an illustration of the expected current distribution rather than a participant-specific representation of the electric field experienced by each individual.

A key contribution of the current study is the distinction between contrast sensitivity and contrast appearance. Previous studies examining the effects of tDCS on contrast processing have primarily focused on detection thresholds or contrast sensitivity, yielding mixed results.^20^^,^^21^^,^^22^^,^^23^^,^^24^^,^^25^ In contrast, our findings demonstrate that anodal stimulation produces a systematic shift in perceived contrast, as indexed by the PSE in a 2AFC appearance judgment task. This distinction is important because changes in threshold-level performance can arise from non-sensory factors, such as external noise filtering, internal noise reduction,^30^^,^^31^ or shifting decision criteria, whereas changes in appearance imply a fundamental alteration in the neural representation of stimulus strength. Under the stimulation parameters and behavioral paradigm employed here, our results are consistent with the hypothesis that anodal tDCS preferentially influences suprathreshold contrast appearance.

Although no statistically significant change in psychometric slope was detected, the present data do not exclude smaller effects on discrimination precision that may require larger samples to detect. If cortical stimulation primarily alters the effective strength of neural responses to suprathreshold stimuli, changes in perceived contrast may therefore be observed even when detection thresholds remain relatively unchanged. Differences in experimental paradigms, stimulus contrast, and baseline performance may contribute to the heterogeneous findings reported previously. Accordingly, the present findings should not be interpreted as implying that occipital tDCS invariably alters visual perception, but rather that contrast appearance may provide a particularly sensitive behavioral assay for detecting stimulation-induced changes in cortical processing under appropriate experimental conditions. Although further studies directly comparing threshold and appearance measures within the same observers are needed, the present results provide a potential framework for understanding these apparently inconsistent findings.

The present findings also provide new insight into the relationship between neuromodulation and attentional modulation of visual perception. The effect of anodal tDCS observed in the current study closely parallels the well-established effects of spatial attention on contrast perception. A large body of work has shown that covert attention modulates contrast gain or neural response gain resulting in improvement in detection of threshold-level stimuli^2^^,^^3^^,^^4^^,^^10^ and alteration in contrast appearance.^7^ The current study demonstrates that anodal tDCS produced a qualitatively similar effect: stimuli presented in the stimulated hemisphere were perceived as higher in contrast, resulting in systematic PSE shifts. This convergence raises the possibility that tDCS-induced modulation of neural excitability may influence perceptual processing by altering the gain mechanisms in early visual cortex. However, both contrast-gain and response-gain mechanisms can modulate perceptual experience of contrast appearance; further research with concurrent neural measurement (e.g., EEG or fMRI) is required to unveil underlying neural mechanisms.

Our findings have broader implications for the use of non-invasive brain stimulation in both basic and applied contexts. From a theoretical perspective, the ability of tDCS to alter perceptual appearance provides causal evidence linking cortical excitability to subjective experience. From an applied standpoint, such modulation may be relevant for interventions aimed at enhancing visual function or compensating for perceptual deficits. The present results also underscore the importance of carefully distinguishing between different levels of analysis—sensitivity, decision, and appearance—when interpreting the effects of neuromodulation. Without such distinctions, changes in perceptual reports could be misattributed to improvements in sensory processing when they in fact reflect shifts in subjective experience.

In summary, the present study provides clear evidence that anodal tDCS over occipital cortex modulates the perceived contrast of visual stimuli in a spatially specific and configuration-dependent manner. These findings extend previous work on tDCS and visual perception by demonstrating a direct effect on appearance, and reveal a striking convergence with the perceptual consequences of spatial attention. Together, our findings highlight the utility of combining psychophysical paradigms with neuromodulation to uncover the causal relationship between neural excitability and perceptual experience.

### Limitations of the study

Several limitations should be considered. First, although the sample size was modest, the effects were highly consistent across observers and associated with large effect sizes. Second, stimulation was restricted to the right occipital cortex. Although the configuration-dependent reversal in PSE argues against a fixed left-right visual field bias, future studies should determine whether comparable effects are obtained following left occipital or bilateral stimulation, thereby establishing the generality of the present findings across hemispheres. Third, the baseline block always preceded stimulation to avoid potential carry-over effects of tDCS. Although this design cannot fully eliminate order-related influences, the absence of comparable shifts during sham stimulation argues against a purely time-dependent account of the observed effects. Fourth, eye movements were not monitored during the experiment. Although the brief stimulus duration (50 ms) makes overt saccades during stimulus presentation highly unlikely, fixation stability was assumed rather than directly verified. Consequently, we cannot completely exclude subtle fixation instability or microsaccade-related influences on the measured responses. Future studies incorporating eye tracking will help confirm fixation maintenance and further strengthen the interpretation of stimulation-induced changes in perceived contrast. Lastly, the sample was not balanced by sex/gender and included only two male participants. The study was therefore not designed or sufficiently powered to evaluate sex- or gender-related differences in the effects of tDCS, which may limit the generalizability of the findings across sex/gender groups.

## Resource availability

### Lead contact

Further information and requests for resources and reagents should be directed to and will be fulfilled by the lead contact, Sang Wook Hong (shong6@fau.edu).

### Materials availability

This study did not generate new reagents.

### Data and code availability

• Newly generated behavioral dataset has been deposited to Mendeley repository. DOI is listed in the key resources table.
• All source code used for data analysis has been deposited to a Mendeley repository. DOI is listed in the key resources table
• All additional information required to reanalyze the data reported in this study is available from the lead contact upon reasonable request.

## Acknowledgments

The author(s) declared that financial support was received for this work and/or its publication. This work was supported by Daqscribe under Grant AWD-003232.

## Author contributions

S.W.H.: conceptualization, funding acquisition, methodology, project administration, supervision, validation, and writing – review and editing. Y.Y.: conceptualization, data curation, formal analysis, investigation, methodology, visualization, and writing – original draft.

## Declaration of interests

The authors declare no competing interests.

## Declaration of generative AI and AI-assisted technologies in the manuscript preparation process

During the preparation of this work the authors used ChatGPT Plus (OpenAI) in order to assist with language editing, manuscript restructuring and formatting. After using this tool/service, the authors reviewed and edited the content as needed and take full responsibility for the content of the published article.

## STAR★Methods

### Key resources table

**Table:** 

REAGENT or RESOURCE	SOURCE	IDENTIFIER
**Deposited data**		

De-identified behavior datasets and analysis code	Mendeley Data	https://data.mendeley.com/datasets/n4s8c4s9cd/1

**Software and algorithms**		

MATLAB R2022a	MathWorks	https://www.mathworks.com
Psychophysics Toolbox Version 3 (PTB-3)	Brainard^32^ Pelli^33^	https://psychtoolbox.org
psignifit3	Schütt et al.^27^	https://github.com/wichmann-lab/psignifit
HD-Explore™ software	Soterix Medical	https://soterixmedical.com/research/hd-explore
Behavioral data analysis code	This paper	https://data.mendeley.com/datasets/n4s8c4s9cd/1

**Other**		

MxN-5 transcranial electrical stimulator	Soterix Medical	Model: MxN-5
Carbon rubber electrodes (3 × 5 cm)	Soterix Medical	N/A
Minolta LS-110 photometer	Konica Minolta	Model: LS-110
LCD monitor (1920 × 1080, 60 Hz)	ASUS	Model: ROG SWIFT 24.5 TFT

### Experimental model and study participant details

#### Human participants

Ten adult participants from Florida Atlantic University, including one of the authors, participated in the experiment. Participants ranged in age from 20 to 53 years and include eight females and two males. No additional demographic information was collected. All participants had normal or corrected-to-normal vision based on the self-report and were unaware of the experiment’s purpose, except for the author participant. All participants completed both the anodal tDCS and sham stimulation conditions on separate days, with sessions separated by at least two days. Participants confirmed that they had no metal implants in the head, no implanted electronic devices, no history of neurological disorders or head injuries, no claustrophobia, were not pregnant, and were not currently taking psychoactive medication. All the participants provided written informed consent form prior to the experiment. The study was approved by the Florida Atlantic University’s Institutional Review Board (IRB2402051) and was conducted in accordance with the approved protocol and applicable institutional guidelines. Participant sex/gender was recorded but was not included as a factor in the statistical analyses because the sample included only two male participants and was not designed or powered to evaluate sex- or gender-related differences in the effects of tDCS.

### Method details

#### Apparatus

Stimuli were generated using MATLAB (MathWorks) with Psychophysics Toolbox (PTB-3) and presented on a 1920 × 1080 display (60 Hz). Participants were seated at a viewing distance of 100 cm with head position stabilized using a chinrest. Background luminance was set to 59.58 cd/m^2^ and was gamma-corrected using a photometer (Minolta LS-110). Transcranial direct current stimulation (tDCS) was delivered using a Soterix MxN-5 system with carbon rubber electrodes (size: 3cm x 5 cm). Anodal stimulation was applied over the right occipital cortex (O2, 10–20 system), and the cathodal electrode was positioned over the fronto-central site (FCz). The maximum electric field intensity went up to 0.49 V/m (Figure 1C), modeled by HD-Explore^TM^ (software provided by Soterix Medical).

#### Stimuli and task

To measure the perceived contrast appearance, we adopted experimental procedure from Carrasco et al. (2004).^7^ The procedure for each trial is described in Figure 1A. At the beginning of each trial, a fixation dot was presented in the middle of the display monitor for 500 msec, which was followed by a cue (a slightly larger black dot than the fixation dot) presentation for 67 msec. The cue was presented at the same location as the fixation so that it could alert participants for upcoming target stimuli presentation while forcing eye fixation to the central location. After 50 msec of blank period (inter stimuli interval between the cue and target stimuli), two oriented Gabor patches were presented the left and right side of the fixation dot. Each Gabor patch was created by convoluting a 2 cycle/deg sinewave grating (oriented ±45° from vertical orientation) with a gaussian envelope and subtended 2-degree visual angle. The location of each Gabor patch (center of the patch) was 4-degree from the fixation (left and right) and 1.5-degree lower than the fixation. The contrast of the test stimuli was varied from 8 to 30% Michelson contrast with 9 log steps (8%, 9.43%, 11.13%, 13.13%, 15.49%, 18.28%, 21.56%, 25.43%, 30%). The contrast of the standard stimulus was set to 15.49% which was the same as 5^th^ contrast of the test stimuli. The test and standard stimuli randomly presented left and right side of fixation for 50 msec, and the order of test stimuli contrast was randomized. Participants were instructed to report the orientation of the Gabor patch that appeared high contrast using designated keys. Following Carrasco et al. (2004),^7^ two sets of two keys (one for left-tilt orientation, the other for right-tilt orientation) were used.

#### Experimental design and stimulation protocol

A single block (pre-stimulation block and stimulation block) was composed of 360 trials (9 test contrast X 2 standard stimulus locations X 20 repetitions). A break was provided every 60 trials, and participants could resume by pressing space bar after short break. In a single session, we first measured the point of subjective equality (PSE) without brain stimulation as a baseline, that indicates which test contrast subjectively appears the same as standard contrast. Ten minutes after the completion of the pre-stimulation block, we measured the PSE while applying 2mA tDCS on the right hemisphere occipital cortex (anodal O2 electrode location on 10-20 EEG system, Figure 1B) in tDCS condition. The order of the blocks was fixed to avoid contamination in baseline measurement from the off-line tDCS effect. The cathodal electrode was placed on FCz electrode location on 10-20 system following conventional montage.^21^^,^^26^^,^^34^ A ground channel was place on the forehead to stabilize low impedance. Once the tDCS session began, the electric current increased to 2mA over 30 seconds and participants were instructed to close their eyes and to rest for 2 minutes, which was estimated duration for the full effect of tDCS. TDCS was applied during the entire stimulation block, approximately taking 10 to 15 minutes to complete, then the stimulator was turned off with ramping down current intensity over 30 seconds. In sham stimulation condition, the electrodes set up was identical to the anodal stimulation condition, but the electric current was ramped up and down over 60 seconds at the beginning and end of the session. Participants reported subtle tingling sensation at the beginning in both sessions. The order of tDCS session and sham stimulation session was randomly assigned to individual participant in different days (at least 2 days apart). The study used a single-blind design in which participants, but not the experimenter, were blind to stimulation condition.

### Quantification and statistical analysis

Psychometric functions were fit separately to each participant’s data for each stimulation condition (anodal and sham), stimulus configuration (LTRS and LSRT), and time point (pre-stimulation and stimulation) using the Bayesian framework implemented in psignifit^27^ (version 3) in MATLAB (MathWorks). The point of subjective equality (PSE) was estimated from each fitted psychometric function as the test contrast at which the participant judged the test stimulus to appear higher in contrast than the standard on 50% of trials. Psychometric slope was also obtained from the fitted functions as a measure of discrimination precision around the PSE. Changes in PSE and psychometric slope were calculated within participants as the difference between stimulation and pre-stimulation measurements. For PSE, ΔPSE was defined as PSE_stim_ − PSE_pre_. For the primary analysis, signed ΔPSE values were calculated by reversing the sign of the LTRS ΔPSE and retaining the sign of the LSRT ΔPSE, such that shifts in the predicted direction were positive in both configurations; the two values were then averaged within each participant.

All statistical analyses were conducted in MATLAB R2022a. The sample consisted of 10 participants (*N* = 10), and the participant was the independent experimental unit for all inferential statistical analyses. The primary comparison of signed ΔPSE between anodal and sham stimulation was performed using a two-tailed paired-samples t-test. One-sample t-tests against zero were additionally used to determine whether signed ΔPSE differed from zero within the anodal and sham conditions. Raw ΔPSE values were analyzed using a 2 × 2 repeated-measures ANOVA with Stimulation (anodal, sham) and Configuration (LTRS, LSRT) as within-participant factors, followed by planned paired-samples t-tests comparing configurations within each stimulation condition and stimulation conditions within each configuration. Corresponding analyses of changes in psychometric slope were performed using paired-samples t-tests and a 2 × 2 repeated-measures ANOVA. Sensitivity analyses were also performed for the primary signed ΔPSE comparison after separately excluding the author participant and the apparent outlier identified in Figure 4.

Statistical tests were two-tailed, and statistical significance was defined as *p* < 0.05. Effect sizes are reported as Cohen’s d_z for paired-samples t-tests and partial eta squared (ηp^2^) for repeated-measures ANOVA effects. Where applicable, 95% confidence intervals are reported. In the Results, descriptive values for signed ΔPSE are reported as mean ± SEM. In Figure 3, symbols represent means across participants and error bars represent ±1 SEM. In Figure 4, individual data points represent participants; box plots show the median and interquartile range, and whiskers indicate the range excluding outliers. Exact statistical results, including test statistics, degrees of freedom, *p* values, effect sizes, confidence intervals, and sample sizes where applicable, are reported in the Results and figure legends.
